# Regulatory of Oleuropein on the In Vitro Maturation of Oocytes and the Development of Parthenogenetic Embryos in Sheep

**DOI:** 10.3390/ani15203011

**Published:** 2025-10-17

**Authors:** Yue Zhang, Wenjuan Zhao, Zihao Ma, Zhenghang Li, Zhijiao Liu, Pengcheng Wan, Guangdong Hu

**Affiliations:** 1 College of Animal Science and Technology, Shihezi University, Shihezi 832000, China; zykysha@163.com (Y.Z.); 19131385019@163.com (Z.M.); 13894117372@163.com (Z.L.); 19914332504@163.com (Z.L.); 2 Institute of Animal Husbandry and Veterinary Science, Xinjiang Academy of Agricultural Reclamation Sciences, Shihezi 832000, China; zwj-130@163.com; 3 National Key Laboratory of Genetic Improvement and Healthy Sheep Breeding, Shihezi 832000, China

**Keywords:** sheep, oocyte, early embryo, oxidative stress, oleuropein

## Abstract

**Simple Summary:**

Oleuropein, as an antioxidant, can alleviate the oxidative stress phenomenon during the in vitro culture of sheep oocytes and early embryos, thereby improving embryo quality. This study found that the appropriate addition of oleuropein can increase the maturation rate of oocytes and promote the development of early embryos. Optimizing the embryo culture system is important for improving the success rate and quality of early embryo development.

**Abstract:**

Oleuropein (OLE), as the main effective active component in olive leaves, is a natural cyclic ether terpene polyphenolic compound found in plants of the genus Olea. It has antioxidant, anti-inflammatory and anti-apoptotic properties, and can reduce damage caused by reactive oxygen species. These characteristics indicate that it can enhance the maturation rate of oocytes and the developmental capacity of embryos—two key indicators in animal breeding. This study evaluated the effects of OLE on the in vitro maturation and early embryonic development of sheep oocytes. 20 μM OLE has the best promoting effect on the maturation rate of oocytes, and 30 μM OLE has the best increasing effect on the blastocyst rate. Compared with the control group, glutathione (GSH) level and mitochondrial membrane potential (MMP) level were significantly increased, ROS level was significantly decreased, the expression of antioxidant genes *SOD1* and *GPX3* was significantly elevated, and the expression of anti-apoptotic gene *BCL2* was significantly elevated in the experimental group. In addition, during the in vitro development stage of early embryos, the expression level of the embryo development-related gene *OCT4* significantly increased. The study has shown that OLE can effectively alleviate oxidative stress during in vitro culture, increase oocyte maturation rate and promote embryo development.

## 1. Introduction

In vitro embryo production (IVP) is critical for agricultural production, farming, human fertility medicine, and research on early embryonic development mechanisms [1]. As research on IVP has expanded, significant progress has been made in applying this technology to various animals, such as sheep [2]. Optimizing the embryo culture system is crucial for improving the success rate and quality of early embryonic development [3]. Typically, the IVP process consists of three primary steps: in vitro maturation (IVM) of oocytes, in vitro fertilization (IVF) or parthenogenetic activation, and in vitro culture (IVC) of embryos to the blastocyst stage [4].

IVM is the first step of IVP. The quality of oocytes is crucial for the successful reproduction and the early embryo development of mammals. Current research primarily focuses on elucidating the correlation between oocyte quality and developmental competence of parthenogenetic embryos. A significant challenge in IVM is achieving nuclear and cytoplasmic maturation synchronously, which represents a major limiting factor for subsequent embryo development. The growth and development of oocytes within an in vitro microenvironment simulating follicular fluid, ultimately enabling their capacity for fertilization, is pivotal to the process of in vitro embryo development. However, the intricate biological processes spanning from oocyte maturation to early embryogenesis are vulnerable. Various disruptive factors may induce meiotic arrest or fertilization failure. Studies have indicated that the developmental potential of oocytes and early embryos in vitro culture is generally lower than in vivo environment. A major factor is the balance between the generation and elimination of ROS is disrupted during oocyte collection and in vitro culture. This imbalance leads to oxidative stress, which impairs oocyte maturation and embryonic development [5]. This suggests a substantial gap between in vitro culture systems and the physiological in vivo environment, indicating that both the culture system and culture mode require further optimization. Therefore, taking effective strategies to mitigate oxidative stress is crucial for improving the efficiency of oocyte maturation and enhancing embryonic developmental competence.

OLE is the primary active component isolated from Ilex pubescent var. kwangsiensis Hand.-Mazz [6]. As an iridoid phenolic compound, it exhibits diverse pharmacological properties including antitumor [7], anti-inflammatory [8], antioxidant [9], detoxification [10], hypoglycemic [11], antihypertensive [12], hypolipidemic [13], and organ-protective effects [14]. Researchers have demonstrated that OLE mitigates oxidative stress-induced cellular damage by suppressing ROS levels and restoring mitochondrial homeostasis. Furthermore, OLE can regulate the TLR4/MAPK and Nrf2 signaling pathways to inhibit oxidative stress and excessive autophagy [15]. While previous studies on oleuropein predominantly focused on anticancer potential, few have explored on animal oocytes. The present study aims to investigate the effects of OLE on in vitro maturation of oocytes and early embryonic development in sheep, thereby providing theoretical support for optimizing embryo culture systems.

## 2. Materials and Methods

### 2.1. Materials

Fetal bovine serum (FBS), MEM-NEAA (100×), GlutaMAX (100×),BME-EAA (50×) and sodium pyruvate (100 mM) were purchased from Gibco (Waltham, MA, USA). Bovine serum albumin (BSA) was purchased from GPC (Beijing, China). TCM199 basal medium was purchased from Pricella (Wuhan, China). The Enhanced Mitochondrial Membrane Potential Assay Kit (JC-1), CellTracker Blue dye (CMF2HC) and ROS Assay Kit (DCFH-DA) were purchased from Beyotime (Haimen, China). Follicle-stimulating hormone (FSH) for injection and luteinizing hormone (LH) for injection were purchased from Solarbio (Beijing, China). The HiFiScript cDNA Synthesis Kit and 2× UltraSYBR Mixture were purchased from CWBIO (Beijing, China). TRIzol was purchased from Ambion (Austin, TX, USA). OLE was purchased from TargetMol (Boston, MA, USA).

### 2.2. Oocyte Collection and In Vitro Maturation

The Hu sheep ovaries were collected by a veterinarian from the local slaughterhouse and transported to the laboratory within 2 h in 0.9% physiological saline maintained at 37 °C. Excess tissue was carefully removed, and the ovaries were rinsed three times with warm physiological saline (37 °C). Follicles were punctured using surgical blades in oocyte collection medium to release cumulus-oocyte complexes (COCs), which deposit in the oocyte retrieval fluid at 37 °C. Only COCs with a homogeneous ooplasm surrounded by at least three layers of compact cumulus cells (corresponding to Grade A and B) were selected. Oocytes were collected under a stereomicroscope (Nikon SMZ800, Tokyo, Japan). The COCs were washed three times in H-TCM199 supplemented with 1% FBS. For each experimental group, 50–80 COCs were placed in four-well plates containing 500 μL of TCM199 medium covered with mineral oil. The cultures were maintained for 24 h in a humidified incubator at 38.5 °C under 5% CO_2_. The TCM199 maturation medium consisted of Earle’s balanced salts, 10% FBS, 1 mM sodium pyruvate, 1× (2 mM) GlutaMAX, 5 μg/mL Folltropin, 20 ng/mL estradiol, and 200 μg/mL gentamicin. The COCs were cultured for 24 h in maturation medium containing 0, 10, 20, and 30 μM OLE. Multiple OLE concentrations were detected to evaluate the optimal dose for supporting the maturation of sheep oocytes in vitro.

### 2.3. Parthenogenetic Activation and Embryo Culture

After 24 h of in vitro culture, oocytes were transferred to medium containing 1 mg/mL hyaluronidase and gently pipetted to remove cumulus cells. The denuded oocytes were washed three times in HEPES-SOF, then activated in HEPES-SOF containing 5% ethanol for 5 min. Following activation, oocytes were washed three times with HEPES-SOF and cultured in 2 mM 6-DMAP for 4 h. After three additional washes with HEPES-SOF solution, the activated oocytes were placed in embryo culture medium and maintained at 38.5 °C under 5% CO_2_ in saturated humidity for 7 days. Cleavage rates were assessed at 48 h post-activation. The culture medium was refreshed every 48 h to maintain optimal nutrient conditions throughout the embryo development period.

### 2.4. Granule Cell Expansion Assessment and Calculation of Maturation Rate, Cleavage Rate, and Blastocyst Rate

The release of the first polar body (PB1) and the expansion of cumulus cells were observed under a microscope (Olympus BX53, Tokyo, Japan) to calculate the oocyte maturation rate. During embryo development, the cleavage rate was determined at 48 h and the blastocyst rate was assessed at 7 days.

### 2.5. ROS and GSH Detection

To evaluate the levels of ROS and GSH in oocytes, denuded oocytes were incubated with 500 μL of fluorescent dye solution containing 10 μM DCFH-DA for ROS detection and 20 μM CMF2HC for GSH measurement. The incubation was conducted at 38.5 °C under 5% CO_2_ in complete darkness for 30 min. Following incubation, oocytes were washed three times with DPBS and immediately analyzed using fluorescence microscopy (Leica DMi8, Wetzlar, Germany). The fluorescence intensity was quantified using ImageJ 13.0 software with identical exposure settings across all samples.

### 2.6. Mitochondrial Membrane Potential Detection

Mitochondrial membrane potential (ΔΨm) was evaluated using JC-1. The JC-1 working solution was prepared by diluting 5 μL of JC-1 (200×) with 1 mL of JC-1 staining buffer. Randomly selected mature oocytes and 8-cell stage embryos were incubated in the JC-1 staining solution for 30 min at 38.5 °C under 5% CO_2_ in a saturated humid atmosphere. Following incubation, samples were immediately observed under a fluorescence inverted microscope (Leica DMi8, Wetzlar, Germany).

### 2.7. Quantitative RT-PCR

Total RNA was extracted from 50 sheep oocytes using RNAprep Pure Micro Kit (Tiangen, Beijing). cDNA was synthesized using HiFiScript cDNA Synthesis Kit. qPCR was performed with UltraSYBR Mixture. Gene expression levels were calculated using the 2^−ΔΔCt^ method with β-actin as internal control. Primer sequences are shown in Table 1.

### 2.8. Statistical Analysis

All data were analyzed by one-way ANOVA using GraphPad Prism 8.0 software, with t-tests for comparisons between two groups. Fluorescence intensity was quantified using ImageJ. Results are expressed as mean ± standard deviation from at least three independent experiments. Statistical significance is defined as follows: *p* > 0.05 (not significant), *p* < 0.05 (significant, *), and *p* < 0.01 (highly significant, **); different superscript letters in the table indicate significant differences.

## 3. Results

### 3.1. Effects of Different OLE Concentrations on In Vitro Maturation of Sheep Oocytes

**Figure 1:**
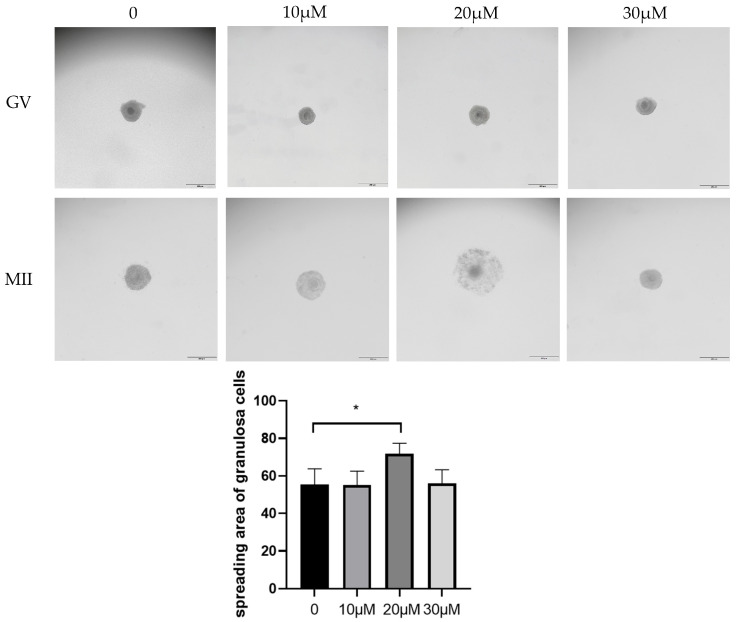
Effect of OLE on cumulus cells expansion in sheep oocytes. * denotes *p* < 0.05.

To determine the optimal OLE concentration for sheep oocyte maturation, different concentrations of OLE (0, 10, 20 and 30 μM) were tested. Oocyte quality was evaluated based on cumulus cell expansion and PB1 extrusion rate, with 10 μM, 20 μM and 30 μM as experimental groups and no OLE addition as control group. Compared with the control group, the 20 μM OLE treatment group showed significantly better expansion effect (*p* < 0.05) (Figure 1). The maturation rate of oocytes in the 20 μM treatment group was also significantly higher than that in the control group (*p* < 0.05) (Table 2). These results indicate that OLE can improve the maturation rate of sheep oocytes, and the optimal concentration was determined to be 20 μM.

### 3.2. Effects of OLE on Oxidative Stress Levels in Sheep Oocytes

**Figure 2:**
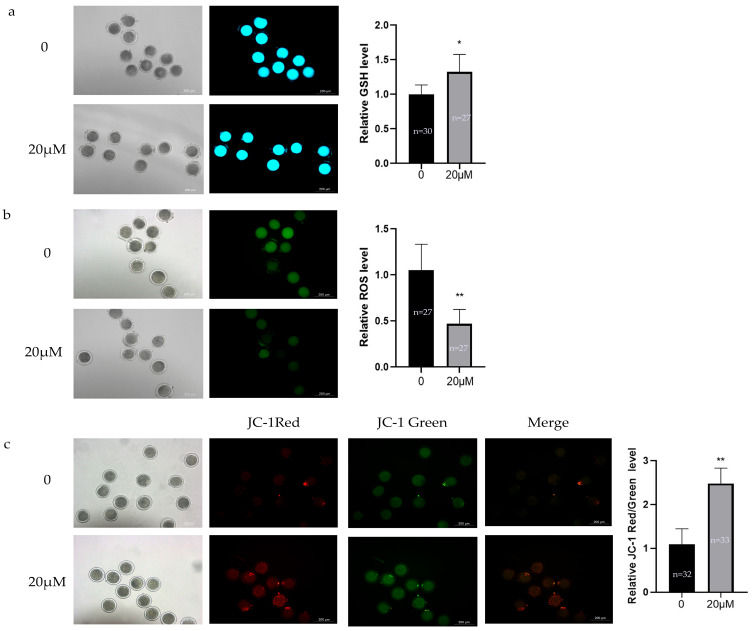
Effects of OLE on oxidative stress levels in sheep oocytes: (**a**) Effect of OLE on GSH levels in sheep oocytes; (**b**) Effect of OLE on ROS levels in sheep oocytes; (**c**) Effect of OLE on MMP levels in sheep oocytes. * denotes *p* < 0.05, ** denotes *p* < 0.01.

To evaluate the antioxidant effects of OLE on in vitro matured sheep oocytes, GSH, ROS and mitochondrial membrane potential levels were assessed from both control and 20 μM OLE-treated groups. As shown in Figure 2, compared with controls, the 20 μM OLE group exhibited significantly higher GSH levels (*p* < 0.05) (Figure 2a), significantly lower ROS levels (*p* < 0.01) (Figure 2b), and a significantly increased mitochondrial membrane potential (*p* < 0.01) (Figure 2c). These results indicate that OLE treatment reduced mitochondrial oxidative stress and enhanced mitochondrial function.

### 3.3. Effects of OLE on Antioxidant-Related and Antiapoptosis-Related Genes in Sheep Oocytes

**Figure 3:**
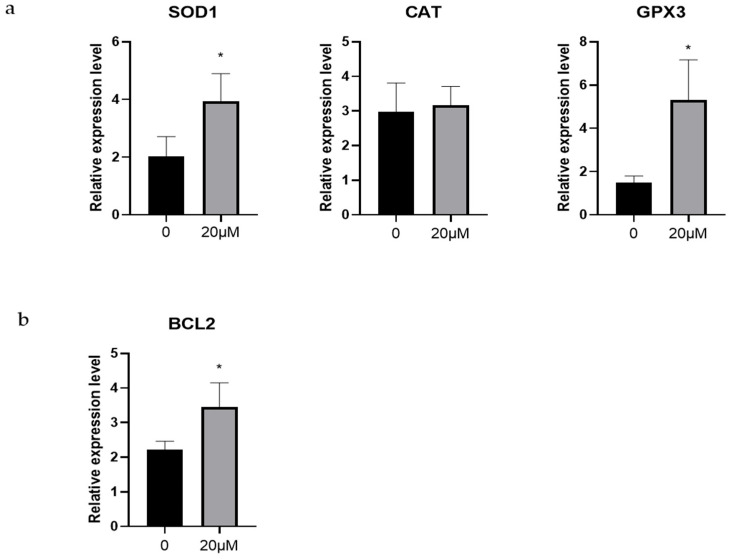
Effects of OLE on antioxidant-related and anti-apoptotic genes in sheep oocytes: (**a**) Effects of OLE on antioxidant-related genes in sheep oocytes; (**b**) Effects of OLE on anti-apoptotic genes in sheep oocytes. * denotes *p* < 0.05.

To investigate the antioxidant effect of OLE during in vitro maturation of sheep oocytes, we measured the expression levels of *SOD1*, *CAT*, and *GPX3*. As shown in Figure 3, the 20 μM OLE treatment group showed significantly increased expression of *SOD1* and *GPX3* in oocytes (*p* < 0.05). In contrast, the expression level of *CAT* is no significant change (*p* > 0.05) (Figure 3a).

In addition, we evaluated the expression of the anti-apoptotic gene *BCL2*. The results indicated that *BCL2* expression was significantly higher in the 20 μM OLE treatment group than in the control group (*p* < 0.01) (Figure 3b).

### 3.4. Effects of Different OLE Concentrations on Early Embryonic Development

**Figure 4:**
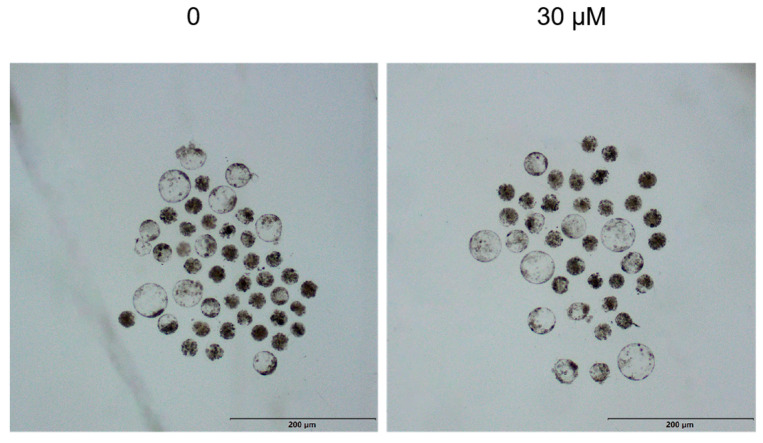
Effects of OLE treatment on the development of parthenogenetically activated embryos.

We examined the effect of different OLE concentrations on the blastocyst rate after parthenogenetic activation of oocytes. The results showed that the 30 μM OLE treatment group have a significantly higher blastocyst rate compared to the control group (*p* < 0.05) (Table 3) (Figure 4).

### 3.5. Effects of OLE on Oxidative Stress Levels in Sheep Early Embryos

**Figure 5:**
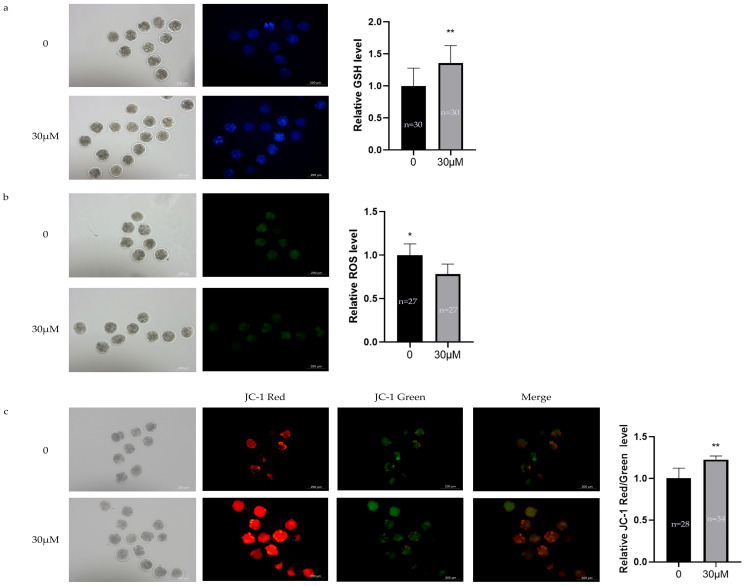
Effects of OLE on oxidative stress levels in sheep early embryos: (**a**) Effect of OLE on GSH levels in sheep early embryos; (**b**) Effect of OLE on ROS levels in sheep early embryos; (**c**) Effect of OLE on MMP levels in sheep early embryos. * denotes *p* < 0.05, ** denotes *p* < 0.01.

As shown in Figure 5, compared with the control group, the 30 μM OLE treatment significantly reduced ROS levels (*p* < 0.05) (Figure 5b), significantly increased GSH levels (*p* < 0.01) (Figure 5a) and MMP levels (*p* < 0.01) (Figure 5c).

### 3.6. Effects of OLE on Antioxidant-Related, Antiapoptosis-Related and Early Embryonic Development-Related Genes in Sheep Early Embryos

**Figure 6:**
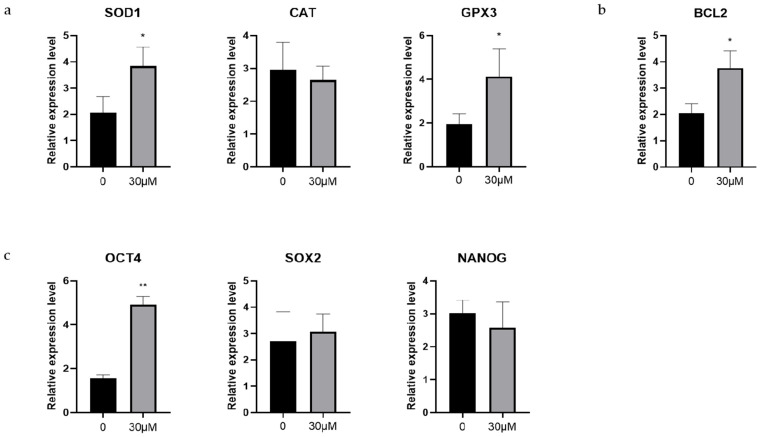
Effects of OLE on antioxidant-related, antiapoptosis-related and early embryonic development-related genes in sheep early embryos; (**a**) Effects of OLE on antioxidant-related genes in sheep early embryos; (**b**) Effects of OLE on antiapoptosis-related genes in sheep early embryos; (**c**) Effects of OLE on early embryonic development-related genes in sheep early embryos. * denotes *p* < 0.05, ** denotes *p* < 0.01.

The mRNA expression levels of genes related to early embryonic development were measured in sheep embryos at the 8-cell stage using RT-qPCR. Among the antioxidant genes, the 30 μM OLE treatment group showed significantly higher expression of *SOD1* compared to the control group (*p* < 0.05), while the expression levels of *CAT* and *GPX3* showed no significant changes (*p* > 0.05) (Figure 6a).

We further examined the expression levels of the anti-apoptotic gene *BCL2*. The results indicated that *BCL2* was markedly higher in the 30 μM OLE treatment group than in the control group (*p* < 0.01) (Figure 6b).

In addition, the expression levels of genes associated with early embryonic development were evaluated. The 30 μM OLE group showed markedly higher expression of *OCT4* compared to the control group (*p* < 0.01), while the expression levels of *SOX2* and *NANOG* showed no significant changes (*p* > 0.05) (Figure 6c).

## 4. Discussion

As an economically important livestock species, sheep exhibit an in vitro oocyte maturation rate of 80%; and the resulting blastocyst rate remains below 50%. However, the pregnancy rate for in vitro-produced embryos (approximately 33–54%) is consistently lower than that of their in vivo-derived counterparts, ROS are a significant factor contributing to this phenomenon [16]. Therefore, obtaining high-quality oocytes and early-stage embryos during IVM is particularly important. Previous experiments have studied the effects of various antioxidants on oocytes and early embryos, such as leonurine, resveratrol, and melatonin. However, up to now, there have been no studies on OLE in related experiments. Previous OLE studies have primarily focused on its therapeutic applications for cancer and cardiovascular diseases. This study represents the first investigation of OLE’s effects on sheep oocyte maturation and early embryonic development.

During IVC of oocytes, the presence of oxidative factors and the deficiency of antioxidants from follicles disrupt the balance between the generation and elimination of ROS. This imbalance breaks the dynamic equilibrium between oxidation and antioxidant defense mechanisms, called oxidative stress. Oxidative stress is typically associated with elevated intracellular ROS levels, which play extensive roles in cellular processes [17]. ROS are highly reactive, short-lived molecules endogenously produced, recognized as crucial mediators in cellular metabolism, proliferation, differentiation, immune regulation, and apoptosis [18]. Excessive ROS activates stress response pathways, causing mitochondrial and plasma membrane instability that permits nonspecific small molecules to penetrate membranes, leading to mitochondrial matrix swelling and apoptosis [15]. ROS overload can directly induce protein denaturation, DNA damage, interfere with DNA replication, and disrupt proper protein synthesis. Furthermore, ROS may degrade intercellular substance components and increase membrane permeability, consequently impairing material transport and signal transduction [19]. Collectively, these effects cause oocyte damage or even cell death. Therefore, supplementing antioxidants appropriately during in vitro oocyte culture is a vital approach for improving oocyte quality.

OLE possesses multiple biological activities such as antioxidant, anti-inflammatory, antitumor, antibacterial, antiviral, cardiovascular protection, and antipsychotic effects. Numerous in vitro and in vivo antioxidant model experiments have confirmed that one prominent biological activity of OLE is its strong antioxidant capacity, particularly in free radical scavenging. Jia He [15] found that OLE activates the Nrf2 antioxidant system, promotes Nrf2 expression and nuclear translocation, thereby increasing the expression of downstream antioxidant enzymes HO-1 and NQO1, further reducing ROS generation and enhancing ROS clearance capacity. Antogneli [20] demonstrated that OLE can mediate the upregulation of mitochondrial glyoxalase 2 (mGlo2) through superoxide dismutase 2 (SOD2)-mediated superoxide anion (O2-·) and AKT signaling pathways, namely by inducing apoptosis in lung cancer cells via the superoxide dismutase 2/superoxide anion/protein kinase B/mitochondrial glyoxalase 2 (SOD2/O2-·/AKT/mGlo2) pathway. Gaia Gherardi [21] discovered that OLE specifically acts on mitochondrial calcium uniporter (MCU), thereby promoting mitochondrial energy metabolism and muscle power. Sonia Devi [22] found that OLE can restrain inflammation and restore immune balance by regulating the phosphoinositide 3-kinase (PI3K)-Akt1 signaling pathway. Additionally, it affects the cell death/autophagy axis and enhances the antimalarial effect of artemisinin. Previous experiments have all indicated that OLE exerts a positive regulatory effect on cells. In this experiment, the above results further confirm the antioxidant capacity of OLE, which plays a protective role in oocyte and early embryonic development.

Mitochondria, as the regulatory hub of mammalian oocytes and early embryonic development, play crucial roles in calcium homeostasis, cytoplasmic redox state regulation, and signal transduction. The capacity of mitochondrial ATP supply and demand is considered the most critical factor affecting oocyte fertilization competence and embryonic developmental potential [23]. Mitochondrial dysfunction and decreased mitochondrial membrane potential lead to reduced oocyte maturation rates and impaired early embryonic development [24]. In oocytes, mitochondria participate in ATP production, signal transduction, and apoptosis [25]. The majority of ATP in eukaryotic cells is generated through oxidative phosphorylation in mitochondria [26]. Emma C Spikings [27] found that mtDNA plays an important role in improving the fertilization capacity of porcine oocytes. The regulation of mtDNA replication timing during porcine oocyte maturation is crucial for successful embryonic development. Furthermore, Hongshan Ge [28] discovered that reduced mitochondrial DNA copy numbers can affect embryonic developmental potential.

Superoxide dismutase is the first line of defense against free radicals, accelerating the reaction of superoxide anion O_2_^−^ with itself to produce H_2_O_2_ and O_2_, then H_2_O_2_ generates H_2_O under the action of *CAT* and *GPX3* [29]. Evidence suggests OLE can mediate *SOD1* to improve mitochondrial function [20]. In this study, the expression level of *SOD1* is significantly higher in the 30 μM OLE-treated group than in the control group, which further substantiates this viewpoint.

*OCT4*, *SOX2*, and *NANOG* are involved in maintaining pluripotency and self-renewal in embryonic. Previous studies have confirmed that *OCT4* plays an important role in the development of oocytes and early embryos in pigs [30], horses [31], cattle [32], and mice [33]. *SOX2* is a key transcription regulator during oocyte-to-embryo transition and is essential for the success of this process [34]. Mudan He [35] found that loss of *NANOG* leads to defects in oocyte maturation and increased endoplasmic reticulum stress. Furthermore, studies have shown that *NANOG*, *OCT4*, and *SOX2* compose a core transcriptional network supporting self-renewal in embryonic cells and cooperatively regulate embryonic development [36]. In this study, the expression level of *OCT4* was significantly increased in 30 μM OLE-treated group.

## 5. Conclusions

In summary, maintaining oxidative balance and mitochondrial functional stability is a crucial step for promoting oocyte maturation and early embryonic development. Appropriate supplementation with OLE can effectively upregulate antioxidant-related, antiapoptosis-related and early embryonic development-related gene expression, reduce oxidative stress, and ultimately improve cellular quality and developmental potential. This approach maximizes the utilization of the genetic potential from elite sheep, thereby enhancing overall flock productivity and economic effectiveness. Concurrently, it effectively conserves sheep genetic diversity by preventing the loss of rare alleles and provides crucial technical support for the purification, rejuvenation, and industrial development of indigenous superior breeds.

## Figures and Tables

**Table 1:** Primer sequences for qRT-PCR.

Gene Name	GenBank Accession	Sequence (5′-3′)	Length (bp)
CAT	XM_004016396	F: CCAGCGACCAGATGAAAC R: CGGTCAAAGTGAGCCATT	175
GPX3	XM_015096153	F: AGGAGAAGTCGAAGATGGACTG R: TCAGTAGCTGGCCACGTTGA	156
SOD1	NM_001145185	F: AGGGAGATAAAGTCGTCGTA R: ACAGAGGATTAAAGTGAGGG	129
BAX	XM_027978594.2	F: CATGGGCTGGACATTGGACT R: CCAGATGGTGAGTGAGGCAG	157
BCL2	XM_027960877.2	F: TGGCCTTCTTTGAGTTCGGA R: CGGTTCAGGTACTCGGTCAT	106
β-Actin	XM_004013078.5	F: AGATTATCGCTCCTCCCG R: CTCATCATACTCCTGCTTGCT	110

**Table 2:** Effect of OLE on the PB1 extrusion rate of sheep oocytes.

Group	Oocytes	PB1 Extrusion Rate/%
0	99	54.55 ± 4.83 ^b^
10 μM/L	103	55.34 ± 4.37 ^b^
20 μM/L	105	71.73 ± 3.26 ^a^
30 μM/L	98	56.12 ± 4.19 ^b^

Different superscript letters in the table indicate significant differences.

**Table 3:** Effect of OLE on the blastocyst rate in sheep.

Group	Oocytes	Cleavage Rate/%	Blastocyst Rate/%
0	103	69.57%	42.72 ± 4.37 ^a^
20 μM/L	105	70.00%	44.76 ± 3.42 ^a^
30 μM/L	112	69.23%	49.11 ± 3.07 ^b^
40 μM/L	101	70.37%	38.61 ± 3.36 ^a^

Different superscript letters in the table indicate significant differences.

## Data Availability

The original contributions presented in this study are included in the article. Further inquiries can be directed to the corresponding authors.
